# SNHG3 could promote prostate cancer progression through reducing methionine dependence of PCa cells

**DOI:** 10.1186/s11658-022-00313-z

**Published:** 2022-02-05

**Authors:** Xiaotian Wang, Yongsheng Song, Yaxing Shi, Da Yang, Jiaxing Li, Bo Yin

**Affiliations:** Department of Urology, Shengjing Hospital of China Medical University, No.36 Sanhao Street, Heping District, Shenyang, 110001 Liaoning China

**Keywords:** Prostate cancer, SNHG3, miR-152-3p, SLC7A11, Methionine dependence

## Abstract

In recent years, morbidity and mortality of prostate cancer (PCa) have increased dramatically, while mechanistic understanding of its onset and progression remains unmet. LncRNA SNHG3 has been proved to stimulate malignant progression of multiple cancers, whereas its functional mechanism in PCa needs to be deciphered. In this study, our analysis in the TCGA database revealed high SNHG3 expression in PCa tissue. Further analysis in starBase, TargetScan, and mirDIP databases identified the SNHG3/miR-152-3p/SLC7A11 regulatory axis. FISH was conducted to assess the distribution of SNHG3 in PCa tissue. Dual-luciferase reporter gene and RIP assays confirmed the relationship among the three objects. Next, qRT-PCR and western blot were conducted to measure expression levels of SNHG3, miR-152-3p, and SLC7A11. CCK-8, colony formation, Transwell, and flow cytometry were carried out to assess proliferation, migration, invasion, methionine dependence, apoptosis, and the cell cycle. It was noted that SNHG3 as a molecular sponge of miR-152-3p stimulated proliferation, migration, and invasion, restrained methionine dependence and apoptosis, and affected the cell cycle of PCa cells via targeting SLC7A11. Additionally, we constructed xenograft tumor models in nude mice and confirmed that knockdown of SNHG3 could restrain PCa tumor growth and elevate methionine dependence in vivo. In conclusion, our investigation improved understanding of the molecular mechanism of SNHG3 modulating PCa progression, thereby generating novel insights into clinical therapy for PCa.

**Supplementary Information:**

The online version contains supplementary material available at 10.1186/s11658-022-00313-z.

## Introduction

Prostate cancer (PCa) is prevalent in men over 50 years old, and ranks as the second leading cause of male deaths after lung cancer [1, 2]. Moreover, the aging population leads to a rapid increase in morbidity and mortality of PCa [3]. PCa features a long latency and a tendency to migrate and invade [4]. PCa has no obvious symptoms at the early stage, whereas it may lead to varying symptoms at an advanced stage, such as dysuria, hematuria, waist soreness, and pelvic pain [5]. At present, the latest advances in adjuvant chemotherapy, radiotherapy, and tumorectomy have markedly improved outcomes of PCa patients, but recurrent risk after treatment and distant metastasis make the 5-year survival rate of PCa patients not optimistic [6, 7]. Hence, new therapeutics are in high demand for improvement of prognosis and life quality of patients. A deeper mechanistic understanding of PCa tumorigenesis and progression at the molecular level adds to the growing body of evidence seeking new biomarkers and potential therapeutic targets. Nonetheless, research on the basic molecular mechanism of PCa pathogenesis and progression is still warranted.

Despite not encoding proteins, long non-coding RNAs (lncRNAs) widely participate in aberrantly cellular biological processes [8], such as cancer pathogenesis and progression [9], tumorous energy metabolism [10], osteogenic differentiation of stem cells [11], and diseases related to the immune system [12]. Numerous lncRNAs can modulate onset, progression, and drug resistance of PCa. For example, lncRNA KCNQ1OT1 regulates the miR-211-5p/CHI3L1 axis to stimulate malignant progression of PCa [13]. LncRNA HOXD-AS1 is a modulator of proliferation and chemo-resistance of castration-resistant PCa through modulating WDR5 [14]. Small nucleolar RNA host gene 3 (SNHG3) as a novel oncogenic lncRNA is theorized to have aberrant expression in varying cancers [15]. For instance, SNHG3 stimulates breast cancer cell malignant behaviors through the miR-186-5p/ZEB1 axis [16]. Silencing SNHG3 is a controller of the miR-216a/ZEB1 axis in non-small cell lung cancer (NSCLC) to constrain cancer malignant progression [17]. Nevertheless, there are some unclear aspects of the molecular mechanism of SNHG3 in PCa. Accordingly, we made an inquiry into the impact of SNHG3 on PCa progression.

Recent investigations revealed a common characteristic of cancer cells that is high methionine dependence [18–20]. Methionine dependence refers to the phenomenon that cells fail to grow in medium with homocysteine (Hcy) instead of methionine (Met) [21]. Currently, methionine restriction is proposed to be an avenue for cancer therapy [22], which is also supported by some preclinical models. For instance, in animal models of rhabdomyosarcoma [23], Yoshida sarcoma [24], hepatoma [25], and colorectal carcinoma [26], methionine restriction functions in repressing tumor growth, preventing cell migration and invasion, and prolonging patient survival. Also, methioninase (METase) can degrade methionine and consume systemic methionine to exert anti-tumor effects in the field of pharmacology [27–29]. Preclinical and clinical studies demonstrated that cooperation of methionine restriction and chemotherapeutics is effective in tumor therapy [30–32]. Researchers revealed that multiplex mRNAs are controllers in tumor progression via affecting methionine dependence of tumor cells [19]. Nevertheless, few researchers have been able to draw on complete research into upstream regulatory factors that influence methionine dependence of tumor cells.

In this study, we uncovered increased SNHG3 expression in PCa tissue through bioinformatics analysis, and probed the role and possible molecular mechanism of SNHG3 as a modulator of methionine dependence and biological behaviors in PCa cells. This study contributes to a deeper understanding of the function of SNHG3 in malignant progression of PCa as well as its influence on methionine dependence of tumor cells. Our findings provide the possibility to lay a theoretical groundwork for the exploitation of new avenues for PCa clinical treatment.

## Materials and methods

### Bioinformatics methods

Gene expression HTSeq-Counts data and mature miRNA expression data were obtained from TCGA-PRAD (https://portal.gdc.cancer.gov/), along with relevant clinical profiles. The R package “survival” was used to assess the correlation between SNHG3 and prognosis of PCa patients. The starBase database (http://starbase.sysu.edu.cn/) was utilized to predict miRNAs that interacted with SNHG3. Candidate target miRNA and SNHG3 were subjected to Pearson correlation analysis. miRDB (http://mirdb.org/), TargetScan (http://www.targetscan.org/vert_72/), mirDIP (http://ophid.utoronto.ca/mirDIP/index.jsp#r), miRWalk (http://mirwalk.umm.uni-heidelberg.de/), and starBase (http://starbase.sysu.edu.cn/) databases were used to identify the downstream target gene of the target miRNA. Gene set enrichment analysis (GSEA) (http://www.gsea-msigdb.org/gsea/index.jsp) was utilized to carry out enrichment analysis on mRNA. The R package “edgeR” was utilized for differential analysis on expression profiles of lncRNA, miRNA, and mRNA between normal and tumor groups (log |FC| > 1.0, FDR < 0.05).

### Patients and tissue samples

We collected 40 pairs of PCa tissue and non-tumor adjacent tissue samples from patients who underwent tumorectomy in Shengjing Hospital of China Medical University in 2020–2021. Samples were cryopreserved in a nitrogen canister. The resected samples were identified as PCa based on histopathological examination. Patients did not undergo radiotherapy or chemotherapy before surgery. They were informed about sample collection and provided written informed consent. This project received approval by the Research Ethics Committee of Shengjing Hospital of China Medical University.

### Cell culture

Human normal prostate stromal immortalized cell line WPMY-1 (BNCC100291) and PCa cell lines PC-3 (BNCC337715), Du 145 (BNCC338240), LNCaP (BNCC337703), and 22RV1 (BNCC100161) were bought from BeNa Culture Collection (Shanghai, China). Cells were prepared at 37℃ with 5% CO_2_. Culture medium information was: PC-3, Du 145, WPMY-1 cell lines in DMEM-H (BNCC338068) plus 10% FBS; LNCaP, 22RV1 cell lines in RPMI-1640 plus 10% FBS (BNCC341471). Du 145 cells were passaged in DMEM (Sigma-Aldrich) plus 10% dialyzed FBS (Gemini Bio-Products) in the methionine dependence assay. Control medium was supplemented with 100 µM l-methionine (Sigma-Aldrich), 100 µM l-cysteine (Fisher Scientific), 1.5 µM vitamin B12, and 4 mM l-glutamine. Also, 200 µM dl-homocysteine (Sigma-Aldrich) was added to the Met-Hcy+ medium. For sulfasalazine treatment, sulfasalazine (HY-14655, medchemexpress, USA) was purchased and dissolved in DMSO, and was added to the culture medium at a final concentration of 200 µM for treating PCa cells. The same amount of DMSO was supplemented to the control group for treatment [33].

### Plasmid construction and cell transfection

pcDNA3.1-SNHG3 (oe-SNHG3), sh-SNHG3, miR-152-3p mimic, miR-152-3p inhibitor, pcDNA3.1-SLC7A11 (oe-SLC7A11), and corresponding negative controls (NCs) were accessed from RiboBio (China). Cell transfection was done with Lipofectamine 2000 (Invitrogen, Carlsbad, CA, USA) per specification.

### Real-time quantitative polymerase chain reaction (qRT-PCR)

Total RNA isolation from cells and tissue was conducted with TRIzol reagent (Invitrogen). A spectrophotometer (Bio-tek) was used for quantification. cDNA was synthesized by reverse transcription from 1 µg of total RNA with a PrimeScript RT kit (Perfect Real Time; TaKaRa Biotechnology). A SYBR Green Master Mix Kit (TaKaRa Biotechnology) was employed for qRT-PCR of SNHG3, miR-152-3p, and SLC7A11. SNHG3 and SLC7A11 took GAPDH as the endogenous control, and miR-152-3p took U6 as the endogenous reference. The 2^−ΔΔCt^ method was utilized to analyze data. Primer sequences are presented in Table 1.

**Table 1:** Primer sequences in qRT-PCR

Gene	Forward (5′–3′)	Reverse (5′–3′)
SNHG3	CAGTGGTCGCTTCTTCTCCTT	GGCATGAAATGCACCTCAAT
SLC7A11	GGCTCCATGAACGGTGGTGTG	GCTGGTAGAGGAGTGTGCTTGC
GAPDH	TGACGTGCCGCCTGGAGAAAC	CCGGCATCGAAGGTGGAAGAG
miR-152-3p	TCGGCAGGTCAGTGCATGACAGAA	CTCAACTGGTGTCGTGGA
U6	CTCGCTTCGGCAGCACA	AACGCTTCACGAATTTGCGT

### Western blot

Cell lysis was performed for 30 min in cold radioimmunoprecipitation assay (RIPA) lysis buffer (Keygen Biotech), followed by measurement of protein concentration with a bicinchoninic acid kit (Beyotime). Equal amounts of protein (40 µg) were electrophoresed on SDS-PAGE gel of an appropriate concentration and were transferred to PVDF membrane. Skimmed milk was recommended for membrane block at room temperature for 1.5 h. Then, the membrane was soaked in primary antibody rabbit anti-SLC7A11 (1/10,000, ab175186, Abcam, Cambridge, UK) overnight at 4 °C, and then in secondary antibody goat anti-rabbit IgG H&L (1/20,000, ab97051, Abcam, UK) for 2 h at room temperature. An enhanced chemiluminescence (ECL) kit (Pierce Biotechnology) was applied for development.

### Fluorescence in situ hybridization (FISH)

Cells were inoculated in a 35-mm culture dish and maintained at 37 °C with 5% CO_2_. LNCaP and Du 145 cells were rinsed with PBS and maintained for 15 min in 4% formaldehyde for fixing, they were then subjected to Triton X-100 and dehydrated with ethanol. The Cy3-labeled SNHG3 probe was bought from RiboBio (Guangzhou, China). Following the manufacturer’s guidelines, RNA FISH was conducted 3 times with a Ribo FISH kit (RiboBio RN: R11060.7) [34]. Finally, images were observed under a Zeiss LSM880 fluorescence microscope (Carl Zeiss Microscopy GmbH, Jena, Germany).

### Cell viability and colony formation assay

According to specification, Cell Counting Kit (CCK)-8 kit (APExBio, Houston, TX, USA) was used to assess cell proliferation. Cells were plated in 96-well plates (1 × 10^3^ cells/well) and cultured under routine conditions for an appropriate time (0, 24, 48, 72, and 96 h). Afterwards, 10 µL of CCK-8 solution (Beyotime, Nanjing, Jiangsu, China) was supplemented to the plate for 1.5 h of cell incubation. The optical density (OD) value of each well was assessed at 450 nm with a Synergy HT multi-mode microplate reader (BioTek, Winooski, VT, USA).

In the colony formation assay, cells were plated in a 6-well plate (2 × 10^3^ cells/well) and maintained for 2 weeks under routine conditions. 4% paraformaldehyde and 0.5% crystal violet were added to fix and stain cell colonies (30 min).

### Cell migration and invasion assays

Cells (1 × 10^5^ cells/well) were inoculated into the upper chamber of a Transwell insert with 8 μm pore size (Costar, Cambridge, MA, USA) without or pre-coated with Matrigel (BD, Franklin Lakes, USA) for migration or invasion analysis, respectively. The lower chamber contained 600 µL of medium and 10% FBS. After 24 h of incubation under routine conditions, cells that migrated or invaded into the lower chamber were fixed with methanol, stained with 0.5% crystal violet, and rinsed with PBS (Gibco; Thermo Fisher Scientific, Inc). Finally, cells were counted under a microscope (200×) (ZEISS, Germany).

### Cell apoptosis and cell cycle

After cell harvesting, they were subjected to Annexin V-FITC and propidium iodide (PI) staining per specification (eBioscience, China). Next, flow cytometry was used for analysis of stained cells to determine live cells, early apoptotic cells, and late apoptotic cells (dead cells).

To measure cell cycle distribution, cells in the 6-well plate were obtained at 24 h after serum starvation and were fixed in 75% ethanol at 4 °C overnight. Cells were then treated with RNase A (Sigma-Aldrich) and PI staining (30 min). Flow cytometry was carried out with an ACEA NovoCyte flow cytometer (Agilent Technologies, USA) and was analyzed by flowjo software.

### Dual-luciferase reporter gene assay

The 3ʹ-UTR sequence of SNHG3 or SLC7A11 was amplified from human genomic DNA and each was cloned into the pmirGLO luciferase reporter vector (Promega, USA). The predicted binding sites of miR-152-3p on the 3ʹ-UTR of SNHG3 or SLC7A11 were mutated with a Quick-change site-directed mutagenesis kit (Agilent Technologies, USA). The SNHG3 vector or SLC7A11 vector WT (MUT) and the NC mimic or miR-152-3p mimic were co-transfected into PCa cells. Forty-eight hours later, luciferase activity was assayed with a TransDetect Dual Luciferase (Firefly) Reporter Assay Kit (Transgen, China) per protocol.

### RNA-binding protein immunoprecipitation (RIP)

The RIP kit (Bes5101, BersinBio, China) was recommended for the assay. Cells (1 × 10^7^) were resuspended in lysis buffer. According to the specification, cell lysate was added to RIP buffer and then incubated on ice. An anti-AGO2 antibody (Abcam, Cambridge, UK) was utilized to capture the mixture of AGO2 protein and its bound RNA, with anti-IgG antibody as a control. Immunoprecipitates were treated with DNase I and proteinase K for 20 min at room temperature. qRT-PCR analysis was carried out on coprecipitated RNA.

### Animal experiments

Six male BALB/c nude mice (4 weeks old) were classified into two groups randomly, three in a group, and twelve male Swiss nude mice (8 weeks old, the animal facilities of the Curie Institute, Paris, France) were randomly grouped into four groups, three in a group. After washing Du 145 cells (transfected with sh-NC or sh-SNHG3) with PBS, they were implanted subcutaneously into the axillary area of either side of BALB/c nude mice. Tumor size was assessed with calipers once a week. Tumor volume was estimated by the formula: volume = length × width^2^ × 0.5. After 5 weeks, mice were euthanized, and tumors were excised and weighed. SNHG3 and Ki67 levels in the resected tumors were analyzed by qRT-PCR and immunohistochemistry (IHC). Swiss mice carrying sh-NC and sh-SNHG3 xenotransplants were fed with a normal diet or a diet lacking MET (MET-HCY+ diet, Villemoisson, France) from day 1 for 3 weeks after transplantation, as listed in Table 2. Three weeks later, Swiss mice were euthanized, and tumors were separated and measured. The ratio of the terminal-stage tumor weight in nude mice in the MET-HCY+ diet group and normal diet group was analyzed.

**Table 2:** Feeds formulas

Component	g/100 g
Sugar	49
Lipids	11
Proteins	14
dl-Methionine^a^	0.3
dl-Homocysteine^b^	0.4
Mineral salts	5
Vitamins	0.37

^a^Control diet

^b^MET-HCY+ diet prepared by UAR (Villemoisson, France)

### IHC

Immunostaining was conducted on paraffin-embedded tumor tissue samples of BALB/c nude mice. The sample was cut into slices (thickness: 4 μm). Through streptavidin-peroxidase coupling, Ki67 antibody (Abcam, Cambridge, UK) was taken as the primary antibody, and goat anti-rabbit IgG H&L (Abcam, Cambridge, UK) was utilized as a secondary antibody for IHC assay. Sections were observed under a 400× microscope (ZEISS, Germany).

### Statistical analysis

Statistical analysis was done on GraphPad Prism 6.0 Software (GraphPad Inc., CA, USA). Measurement data were expressed as mean ± standard deviation. Comparison between two groups was made by *t*-test, and comparison among multiple groups was made by analysis of variance (ANOVA). *P *< 0.05 means the difference was statistically significant.

## Results

### Increased SNHG3 in PCa tissue and cells is distributed in cytoplasm

**Fig. 1:**
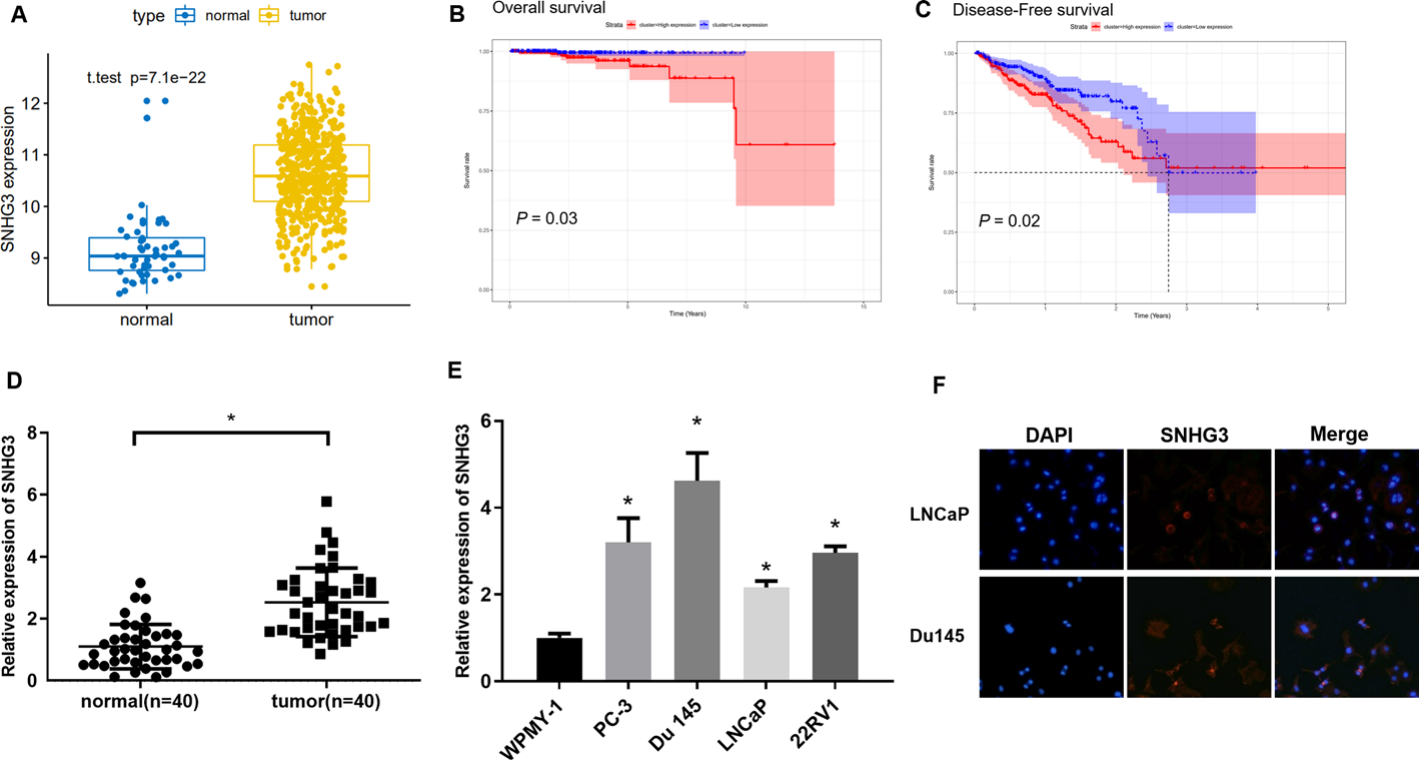
Increased SNHG3 in PCa tissue and cells is distributed in cytoplasm.** A** SNHG3 expression in PCa tissue and non-tumor adjacent tissue in TCGA database. Blue box: normal tissue; Yellow box: tumor tissue; **B**, **C** Kaplan–Meier survival analysis of overall survival and disease-free survival between SNHG3 high expression (red) and low expression (blue) patients in TCGA database; **D** SNHG3 expression in PCa tissue and paired non-tumor adjacent tissue (normal = 40, tumor = 40); **E** qRT-PCR assay of SNHG3 in normal prostate stromal immortalized cells (WPMY-1) and PCa cells (PC-3, Du 145, LNCaP, 22RV1). The experiment was repeated 3 times, *p < 0.05; **F** FISH detected distribution of SNHG3 in PCa cells

At present, SNHG3 can serve as a ceRNA to exacerbate malignant progression of various cancers [35–37], but few studies have been carried out on its mechanism in PCa. Through analyzing TCGA-PRAD gene expression data, we found thatSNHG3 was conspicuously highly expressed in PCa tissue compared to normal prostate tissue (Fig. 1A). Survival analysis revealed that patients with a high SNHG3 level were associated with an unfavorable prognosis and reduced disease-free survival (Fig. 1B, C). qRT-PCR was performed to analyze SNHG3 level in 40 pairs of PCa tissue and non-tumor adjacent tissue. It was found that SNHG3 level in PCa tissue was markedly increased compared to that in non-tumor adjacent tissue. The relationship between SNHG3 and patient’s pathological information was assessed. The results showed that SNHG3 expression was notably correlated with the patient’s disease progression (Fig. 1D, Additional file 1: Table S1). Also, SNHG3 expression was detected in PCa cells. As presented in Fig. 1E, compared with WPMY-1 cells, SNHG3 level was remarkably increased in PCa cell lines (PC-3, Du 145, LNCaP, and 22RV1). Moreover, FISH showed that SNHG3 was mainly distributed in cytoplasm of LNCaP and Du 145 cells (Fig. 1F). These findings illustrated that increased SNHG3 may be implicated in PCa progression.

### SNHG3 hastens proliferation, migration, and invasion, and suppresses apoptosis of PCa cells

**Fig. 2:**
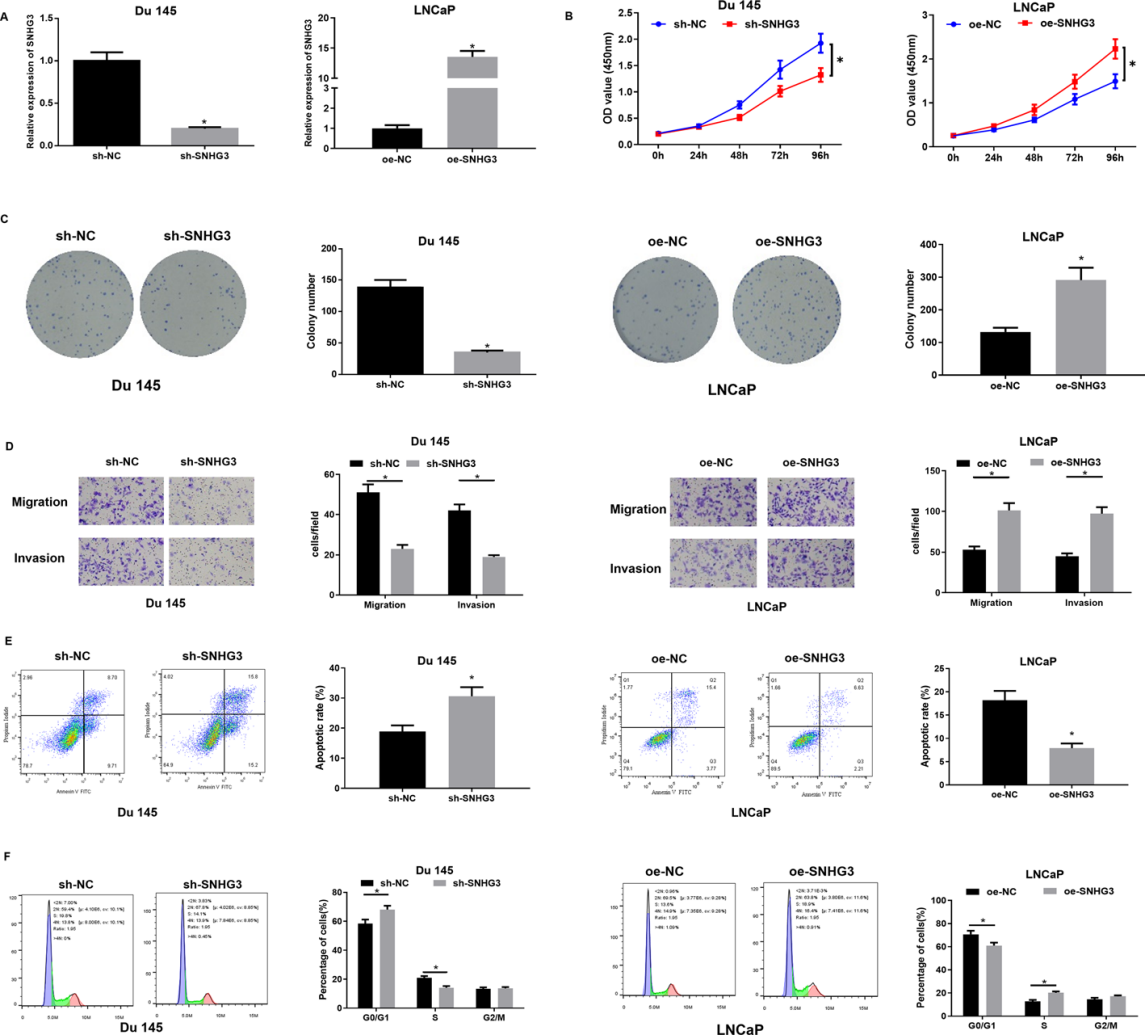
SNHG3 knockdown hampers proliferation, migration, and invasion, and stimulates apoptosis of PCa cells.** A** qRT-PCR assessed SNHG3 expression in PCa cells), *p < 0.05; **B** CCK-8 assay determined proliferation of PCa cells, *p < 0.05; **C** Colony formation assay measured colony formation in PCa cells, *p < 0.05; **D** Transwell assessed migration and invasion of PCa cells, *p < 0.05; **E**, **F** flow cytometry detected apoptosis and cell cycle distribution of cells transfected with sh-SNHG3. All the experiments were repeated 3 times, *p < 0.05

Since SNHG3 level was the highest in Du 145 cells and the lowest in LNCaP cells, we transfected oe-SNHG3 into LNCaP cells and transfected sh-SNHG3 into Du 145 cells to construct PCa cells with overexpressed and silenced SNHG3, thereby evaluating the biological role of SNHG3 in PCa (Fig. 2A). CCK-8 and colony formation assays confirmed cell proliferative changes. As plotted in Fig. 2B, C, SNHG3 knockdown hindered cell proliferation and colony formation, while SNHG3 overexpression markedly fostered proliferative ability of PCa cells. Transwell assay revealed that SNHG3 knockdown remarkably decreased cell migratory and invasive abilities, while SNHG3 overexpression noticeably enhanced these abilities of PCa cells (Fig. 2D). Moreover, flow cytometry was run to assess changes in cell apoptosis and the cell cycle. As illustrated in Fig. 2E, the apoptotic rate of cells in the sh-SNHG3 group was conspicuously higher compared to the control group, while that of cells in the oe-SNHG3 group decreased. Cell cycle data showed that in the PCa cell line, knockdown of SNHG3 notably decreased the S phase cell ratio. The cell cycle was arrested in G0/G1 phase, and overexpression of SNHG3 accelerated cell cycle transition from G0/G1 phase to S phase (Fig. 2F). Hence, SNHG3 exerted a promoting effect on proliferation, migration, and invasion, and had a repressive impact on apoptosis of PCa cells.

### SNHG3 serves as a molecular sponge of miR-152-3p

**Fig. 3:**
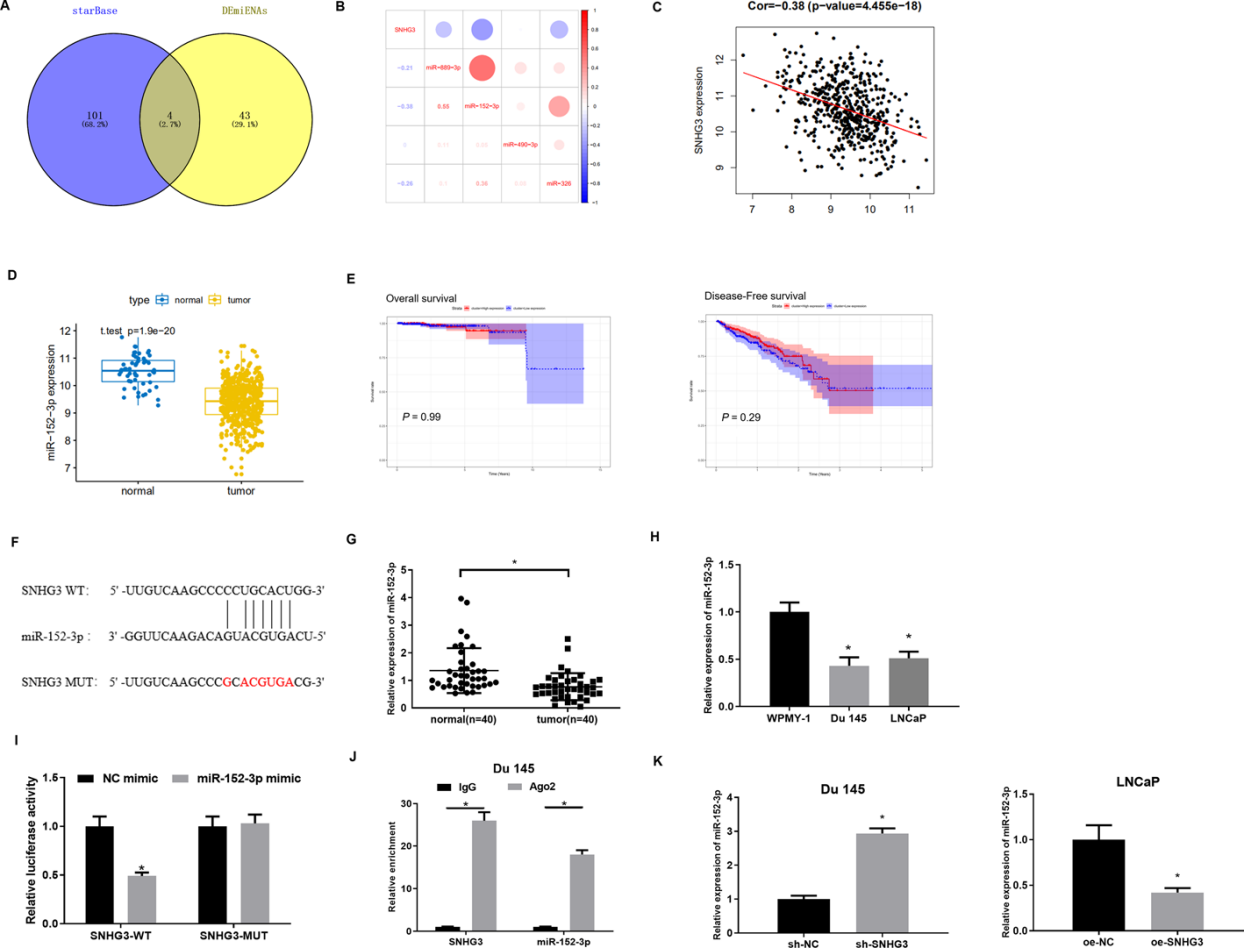
SNHG3 serves as a molecular sponge of miR-152-3p.** A** Venn diagram of predicted target miRNAs and differential miRNAs; **B** heatmap of Pearson correlation analysis of candidate target miRNA and SNHG3; **C** scatter plot of Pearson correlation analysis of miR-152-3p and SNHG3; **D** box plot of miR-152-3p level in normal and tumor groups; **E** Kaplan–Meier survival analysis of overall survival and disease-free survival between miR-152-3p high expression and low expression patients in TCGA database. **F** Binding sequences of SNHG3 and miR-152-3p; **G** miR-152-3p level in PCa clinical tissue and paired non-tumor adjacent tissue (normal = 40, tumor = 40); **H** miR-152-3p expression in PCa cell lines and normal cell line. The experiment was repeated 3 times, *p < 0.05; **I** dual-luciferase assay validated targeted relationship of miR-152-3p and SNHG3 in Du 145 cells. The experiment was repeated 3 times, *p < 0.05; **J** RIP assessed the enrichment of miR-152-3p and SNHG3 in Ago2 immunoprecipitation. The experiment was repeated 3 times, *p < 0.05; **K** miR-152-3p level in Du 145 cells and LNCaP cells with sh-SNHG3 or oe-SNHG3. The experiment was repeated 3 times, *p < 0.05

Firstly, we intersected 47 down-regulated miRNAs obtained by differential analysis with predicted miRNAs that had binding sites in SNHG3 based on an inverse modulatory relationship of lncRNA-miRNA in ceRNA. Four differential miRNAs that had binding sites with SNHG3 were obtained (Fig. 3A). Pearson correlation analysis showed that miR-152-3p was inversely related to SNHG3 and had the highest correlation coefficient (Fig. 3B, C). Difference analysis of miRNA data from TCGA-PRAD displayed that miR-152-3p level was remarkably decreased in PCa tissue (Fig. 3D). Survival analysis showed no significant difference in overall survival or disease-free survival between miR-152-3p high expression and low expression patients (Fig. 3E). There were complementary binding sites in SNHG3 and miR-152-3p (Fig. 3F).

To prove the above prediction, we first assessed miR-152-3p level in PCa tissue and cell lines. It was found that miR-152-3p was markedly reduced in PCa tissue and cell lines. The relationship between miR-152-3p and patient’s pathological information was assessed. The results revealed that miR-152-3p expression was notably correlated with patient’s disease progression (Fig. 3G, H, Additional file 1: Table S2). Next, SNHG3-WT and SNHG3-MUT reporter vectors were established for dual-luciferase assay. The results presented in Fig. 3I revealed that miR-152-3p mimic transfection repressed luciferase activity of the SNHG3-WT group while having no effect on luciferase activity of the SNHG3-MUT group. RIP assay illustrated that immunoprecipitation with Ago2 antibody caused enrichment of miR-152-3p and SNHG3 in Du 145 cells (Fig. 3J), indicating a targeted relationship of miR-152-3p and SNHG3. Transfection of sh-SNHG3 into Du 145 cells and oe-SNHG3 into LNCaP cells was completed to check whether miR-152-3p level is affected by SNHG3 expression. The results of qRT-PCR indicated that miR-152-3p was conspicuously upregulated after transfecting with sh-SNHG3, while it was hindered by transfecting with oe-SNHG3 (Fig. 3K).

### SLC7A11 is the direct target of miR-152-3p

**Fig. 4:**
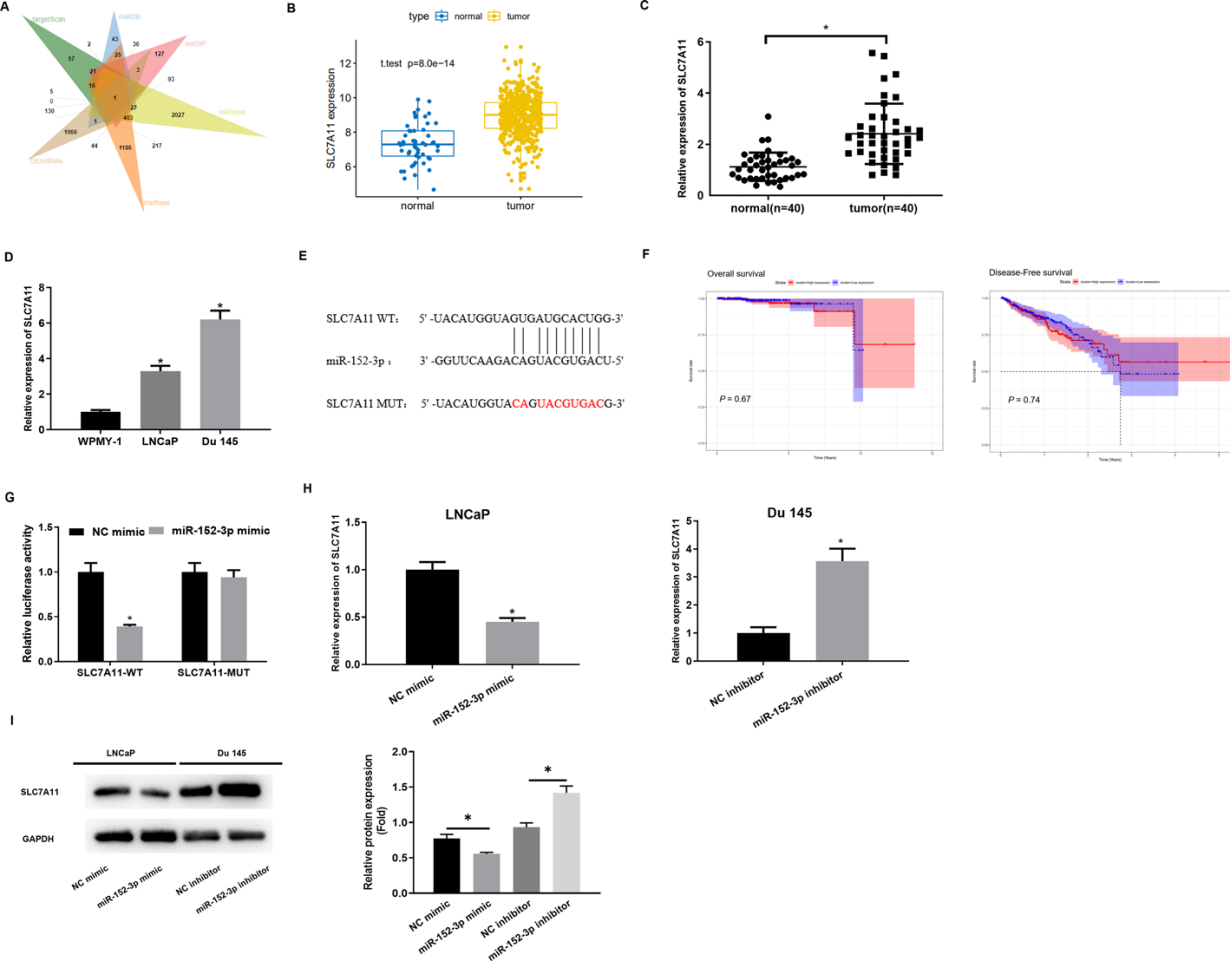
SLC7A11 is the direct target of miR-152-3p.** A** Venn diagram of predicted target mRNAs and differential mRNAs; **B** SLC7A11 expression in TCGA database in normal and tumor groups; **C** SLC7A11 level in PCa clinical tissue and non-tumor adjacent tissue (normal = 40, tumor = 40); **D** SLC7A11 level in PCa and normal cell lines. The experiment was repeated 3 times, *p < 0.05; **E** binding sequence of SLC7A11 and miR-152-3p; **F** Kaplan–Meier survival analysis of overall survival and disease-free survival between SLC7A11 high expression and low expression patients in TCGA database. **G** Dual-luciferase assay confirmed targeted relationship of miR-152-3p and SLC7A11. The experiment was repeated 3 times, *p < 0.05; **H**, **I** SLC7A11 mRNA and protein levels in Du 145 cells after overexpression or inhibition of miR-152-3p. The experiment was repeated 3 times, *p < 0.05

Up-regulated differential mRNAs were intersected with the predicted downstream targets of miR-152-3p to obtain SLC7A11 that could bind to miR-152-3p (Fig. 4A). An analysis of the TCGA database showed that SLC7A11 was notably increased in PCa tissue (Fig. 4B). As such, SLC7A11 level was assayed in PCa tissue and cell lines. SLC7A11 was found to increase in PCa tissue and cell lines. The relationship between SLC7A11 and patient’s pathological information was assessed. The results showed that SLC7A11 expression was notably correlated with the patient’s disease progression (Fig. 4C, D, Additional file 1: Table S3). Then, it was found that miR-152-3p and SLC7A11 had complementary binding sites (Fig. 4E). Survival analysis showed no significant difference in overall survival and disease-free survival between SLC7A11 high expression and low expression patients (Fig. 4F). To further determine targeted relationship of miR-152-3p and SLC7A11, the dual-luciferase assay was performed. As presented in Fig. 4G, luciferase activity of cells with SLC7A11-WT was downregulated by miR-152-3p mimic while that of cells with SLC7A11-MUT was not affected. Meanwhile, qRT-PCR and western blot were utilized to determine whether miR-152-3p overexpression affects SLC7A11 expression. Transfection of miR-152-3p mimic hampered SLC7A11 expression, while SLC7A11 level increased in cells in the miR-152-3p inhibitor group (Fig. 4H, I). Collectively, miR-152-3p constrained SLC7A11 expression in PCa.

### SNHG3 modulates malignant progression of PCa via miR-152-3p/SLC7A11 axis

**Fig. 5:**
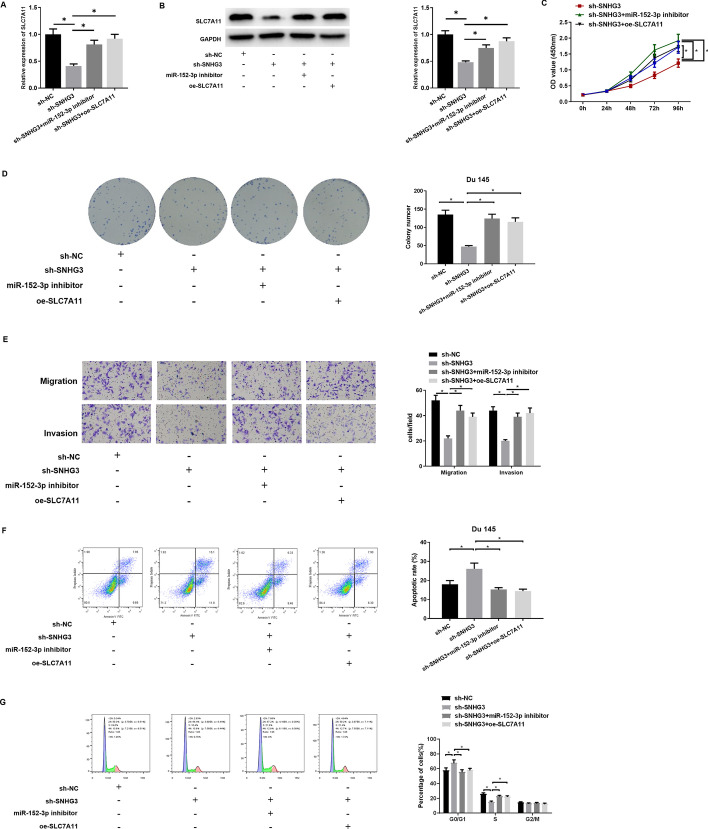
SNHG3 facilitates growth of PCa cells via miR-152-3p/SLC7A11 axis.** A** SCL7A11 mRNA expression in cells of each transfection group (sh-NC, sh-SNHG3, sh-SNHG3 + miR-152-3p inhibitor, sh-SNHG3 + oe-SLC7A11), *p < 0.05; **B** protein expression of SLC7A11 in cells of each transfection group, *p < 0.05; **C** CCK-8 assayed proliferative ability of Du 145 cells, *p < 0.05; **D** colony formation assay detected colony formation of Du 145 cells, *p < 0.05; **E** transwell measured migration and invasion of Du 145 cells, *p < 0.05; **F**, **G** flow cytometry assessed apoptosis and cell cycle progress of Du 145 cells. All the above experiments were repeated 3 times, *p < 0.05

To probe whether SNHG3 miR-152-3p has an oncogenic role via targeting SLC7A11, SNHG3 was knocked down for rescue experiments. qRT-PCR and western blot were conducted to assay mRNA and protein levels of SLC7A11 in each transfection group (Fig. 5A, B). Next, a series of cellular biological assays was carried out on each group. The results presented in Fig. 5C–G illustrated that SNHG3 knockdown could repress proliferation, migration, and invasion, and retard cell cycle progress, while hastening apoptosis of Du 145 cells. However, miR-152-3p knockdown or SLC7A11 overexpression could reverse these inhibitory effects on malignant progression of Du 145 cells. Together our findings demonstrated that SNHG3 fostered PCa progression through modulating the miR-152-3p/SLC7A11 axis.

### SNHG3/miR-152-3p/SLC7A11 modulatory axis can influence methionine dependence of PCa cells

**Fig. 6:**
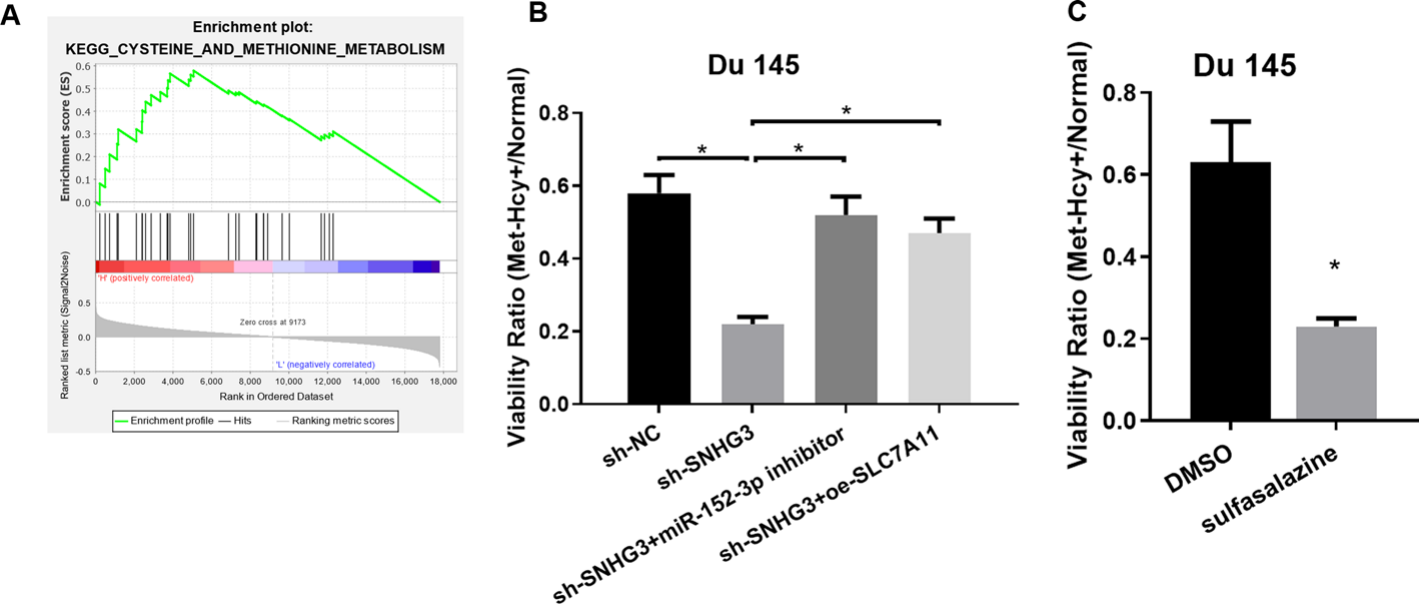
SNHG3/miR-152-3p/SLC7A11 modulatory axis can influence methionine dependence of PCa cells.** A** GSEA enrichment analysis of SLC7A11; **B** CCK-8 measured methionine dependence of PCa cells in each transfection group (sh-NC, sh-SNHG3, sh-SNHG3 + miR-152-3p inhibitor, sh-SNHG3 + oe-SLC7A11). The experiment was repeated 3 times, *p < 0.05. **C** CCK-8 measured methionine dependence changes of PCa cells during sulfasalazine treatment. The experiment was repeated 3 times, *p < 0.05

A study ascertained that decreased SLC7A11 is associated with elevated methionine dependence in breast cancer cells [19]. Through GSEA pathway enrichment analysis, it was found that there was a conspicuous difference in cysteine and methionine metabolism signaling pathway activity between patients with a high SLC7A11 level and patients with a low SLC7A11 level (Fig. 6A). Afterward, Du 145 cells were transfected with sh-NC, sh-SNHG3, sh-SNHG3 + miR-152-3p inhibitor, and sh-SNHG3 + oe-SLC7A11, and they were cultured in normal medium and Met-Hcy+ medium. CCK-8 assay was performed, and the results showed that cell viability of the sh-SNHG3 group in Met-Hcy+ medium and normal medium was markedly lower than that of the sh-NC group, while the ratio of cell viability of sh-SNHG3 + miR-152-3p inhibitor and sh-SNHG3 + oe-SLC7A11 groups in Met-Hcy+ medium and normal medium recovered (Fig. 6B), suggesting that silencing SNHG3 elevated methionine dependence of PCa cells. However, this effect was rescued via silencing miR-152-3p or overexpressing SLC7A11. Hence, SNHG3 could modulate SLC7A11 expression through miR-152-3p and affect methionine dependence of PCa cells. Finally, we selected the SLC7A11 inhibitor sulfasalazine to ascertain whether sulfasalazine can foster methionine dependence of tumor cells. The results demonstrated that methionine dependence of tumor cells was conspicuously increased after sulfasalazine treatment (Fig. 6C). This indicated that sulfasalazine could increase the methionine dependence of tumor cells by repressing SLC7A11.

### SNHG3 influences growth of solid tumor and methionine dependence of PCa in nude mice

**Fig. 7:**
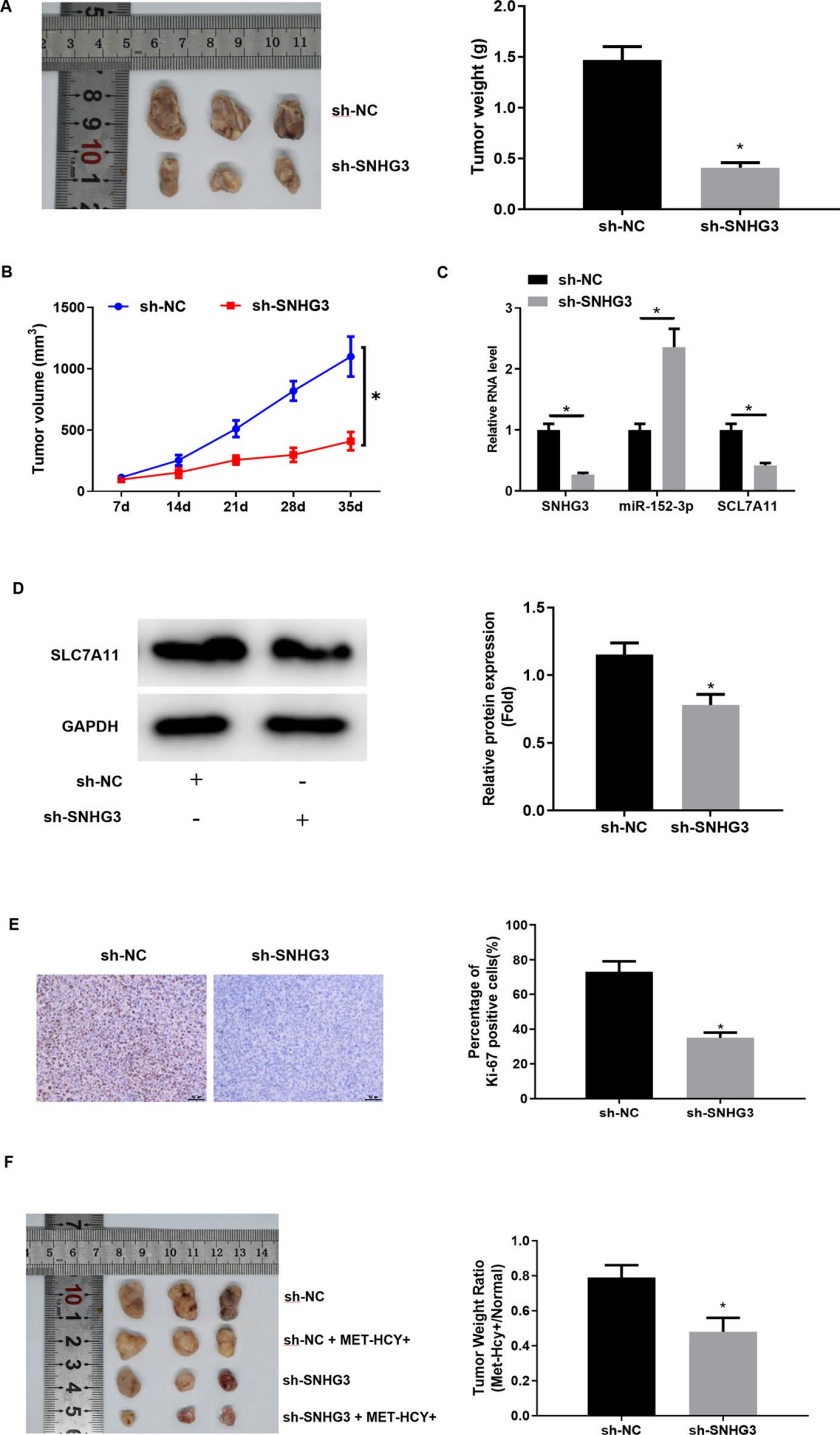
SNHG3 influences growth of solid tumor and methionine dependence of PCa in nude mice.** A** Tumor image and tumor weight dissected from nude mice 35 days later (N = 3, *p < 0.05); **B** tumor growth curve (N = 3, *p < 0.05); **C**, **D** SNHG3, miR-152-3p, and SLC7A11 levels in tumor tissue were assessed via qRT-PCR and western blot. The experiment was repeated 3 times, *p < 0.05; **E** percentage of Ki-67 positive cells in xenogeneic tumor tissue assessed by IHC (scale bar: 50 μm). The experiment was repeated 3 times, *p < 0.05; **F** tumor weight ratio of nude mice at terminal stage with a normal diet or a diet lacking MET (MET-HCY+ diet) (N = 3, *p < 0.05)

We constructed a xenograft mouse model for further analysis of the impact of SNHG3 on growth and methionine dependence of PCa in vivo. Experiments in vivo showed that 35 days later, nude mice tumor volume and weight in the sh-SNHG3 group were notably lower than those in the sh-NC group (Fig. 7A, B). qRT-PCR and western blot assays evaluated SNHG3, miR-152-3p, and SLC7A11 levels in tumor tissue, respectively. The results indicated that levels of SNHG3 and SLC7A11 were conspicuously low while that of miR-152-3p was remarkably high in the sh-SNHG3 group when compared with the sh-NC group (Fig. 7C, D). IHC assay evidenced that compared with subcutaneous tumors in the control group, the proportion of Ki-67 positive cells in subcutaneous tumors in the sh-SNHG3 group was dramatically reduced (Fig. 7E). Hence, SNHG3 knockdown hindered tumor growth of PCa in vivo. Afterward, we compared the tumor weight ratio of mice at the terminal stage with a normal diet or a diet lacking MET (MET-HCY+ diet). As presented in Fig. 7F, the tumor weight ratio of nude mice in vivo in the sh-SNHG3 group was remarkably lower than that in the sh-NC group, indicating that SNHG3 silence could enhance methionine dependence of PCa cells in nude mice. These findings demonstrated that SNHG3 could influence growth of solid tumors and methionine dependence of PCa in nude mice.

## Discussion

At present, numerous lncRNAs are found to be associated with PCa progression. However, various lncRNAs have different effects on the molecular mechanism of PCa progression and malignant phenotype of cancer cells. This issue needs to be addressed. In this study, through the TCGA database, SNHG3 was found to be upregulated in PCa. Through cellular biological experiments, SNHG3 was found to facilitate proliferation, migration, invasion, and cell cycle progress, and hinder cell apoptosis of PCa cells, which was congruous with its function in larynx carcinoma [36] and breast cancer [38]. Moreover, we manifested that SNHG3 knockdown could hinder in vivo PCa tumor growth. Accordingly, we believe that SNHG3 acted as an oncogene in PCa.

LncRNA can modulate downstream miRNA, thereby affecting mRNA expression and biological behaviors of diseases [39]. Researchers have revealed that SNHG3 can be a sponge of miRNA to modulate onset and progression of a variety of cancers. For instance, SNHG3 can serve as a ceRNA to exacerbate colorectal cancer malignant progression through the miR-182-5p/c-Myc axis [35]. SNHG3 serves as a molecular sponge of miR-577 to upregulate SMURF1 level, thus facilitating PCa cell proliferation, migration, and invasion [40]. Furthermore, existing investigations reported that miR-152-3p can influence progression of colon carcinoma [41], gastric cancer [42], and glioma [43]. MiR-152‐3p is also a controller of progression of PCa [44, 45]. In PCa, miR-152-3p serves as a tumor repressor to suppress TMEM97 expression, thus hindering tumor growth [46]. Nonetheless, previous studies have not dealt with the regulatory effect of SNHG3 on miR-152-3p to influence onset and progression of cancer, and relative studies on downstream targets and the modulatory mechanism of miR‐152‐3p in PCa are warranted. In this investigation, through the starBase database, we identified miR-152-3p that was dysregulated in PCa and had binding sites with SNHG3. Their targeted relationship was confirmed through dual-luciferase and RIP assays. Furthermore, we also observed that silencing miR-152-3p restored the repressive impact of SNHG3 knockdown on malignant progression of PCa cells.

In addition, it was demonstrated that SLC7A11 was a downstream target of miR-152-3p and was activated in PCa tissue, showing the same trend in NSCLC [47], cervical cancer [48], and thyroid papillary carcinoma [49]. The targeted relationship of miR-152-3p and SLC7A11 was confirmed via dual-luciferase assay. Rescue experiments were designed for verification. It was found that miR-152-3p silence or forced SLC7A11 expression could rescue the impact of sh-SNHG3 on malignant phenotypes of Du 145 cells. Next, GSEA pathway enrichment analysis revealed that SLC7A11 was mainly enriched in the cysteine and methionine metabolism pathway. It is worth noting that decreased SLC7A11 level is related to increased methionine dependence of breast cancer cells and drug resistance of gastric cancer [19, 50]. Methionine dependence is vital to repress tumor growth and can affect progression of multiple tumors, such as PCa, breast cancer, and colorectal cancer [51, 52]. Mavrakis et al. [53] reported that in MTAP/CDKN2A-deleted cancers, metabolic disorder of methionine leads to PRMT5 dependence of cancer cells. Chen et al. [54] reported that in the methionine metabolic pathway, RUNX3, AMD1 and MSRA genetic variants may be promising predictors for NSCLC patients’ survival. In addition, methionine dependence can affect PCa progression via cell cycle regulation [55]. So far, several preclinical investigations have presented a synergistic action between methionine restriction and varying cytotoxic chemotherapeutic agents [22]. In the past three decades, accumulating experiments have evidenced that methionine restriction can become a supernumerary avenue for cancer therapy [56]. Hence, research on the mechanism of methionine restriction of tumor progression is of great significance to exploring therapeutic regimens for cancers. In this study, we researched the relationship between the SNHG3/miR-152-3p/SLC7A11 axis and methionine dependence of PCa cells. We found that SLC7A11 overexpression could reverse the increase of SNHG3 knockdown on methionine dependence of PCa cells, indicating that SNHG3 modulated methionine dependence of PCa cells via the miR-152-3p/SLC7A11 axis. We used sulfasalazine to treat Du 145 cells. The results showed that sulfasalazine significantly increased the methionine dependence of tumor cells. Thus, we believe that SNHG3 served as a sponge of miR-152-3p to modulate methionine dependence of PCa cells by targeting SLC7A11, thereby facilitating PCa progression. Interestingly, we found that a veterinary clinical study once reported that a golden retriever that had taken sulfasalazine for a long time had unexplained drug adverse reactions and developed liver necrosis. After *s*-adenosyl methionine was treated and sulfasalazine was stopped, it recovered and was discharged, indicating that sulfasalazine may indeed inhibit the metabolism of methionine in vivo [57]. In addition, it has been observed in tumor-related studies that sulfasalazine can inhibit tumor resistance to paclitaxel and improve the efficacy of chemotherapy [33]. Based on the above studies, we believe that SLC7A11 can be used as a tumor therapeutic target, and its inhibitor sulfasalazine can increase the methionine dependence of tumors in vivo and repress tumor growth. Our results were in complete accord with the trend reported in published investigations.

Overall, our study identified the oncogenic role of SNHG3 in PCa. We verified by experiments that SNHG3 acted as a sponge of miR-152-3p to affect the SLC7A11 level, thus modulating methionine dependence of PCa cells and hastening progression of PCa. Our investigation generates novel insights into the role of SNHG3 in PCa and is conducive to diagnostic and therapeutic tool development for PCa management. Since we did not collect complete clinical information of patients, we failed to provide patient OS and DFS data in clinical trials to verify the results predicted through TCGA. We plan to carry out clinical trials to explore the role of SNHG3 in PCa.

## Supplementary Information

**Additional file 1: Table S1.** Correlations between SNHG3 expression and clinicopathological characteristics in prostate cancer. **Table S2.** Correlations between miR-152-3p expression and clinicopathological characteristics in prostate cancer. **Table S3.** Correlations between SLC7A11 expression and clinicopathological characteristics in prostate cancer.

## Data Availability

The data used to support the findings of this study are included within the article. The data and materials in the current study are available from the corresponding author on reasonable request.

## Abbreviations

PCa: Prostate cancer
lncRNAs: Long non-coding RNAs
SNHG3: Small nucleolar RNA host gene 3
NSCLC: Non-small cell lung cancer
Hcy: Homocysteine
Met: Methionine
METase: Methioninase
qRT-PCR: Quantitative polymerase chain reaction
RIPA: Radioimmunoprecipitation assay
ECL: Enhanced chemiluminescence
ANOVA: Analysis of variance

## References

[1] Michaelson MD, Cotter SE, Gargollo PC, Zietman AL, Dahl DM, Smith MR (2008). Management of complications of prostate cancer treatment. CA Cancer J Clin.

[2] D’Elia C, Cerruto MA, Cioffi A, Novella G, Cavalleri S, Artibani W (2014). Upgrading and upstaging in prostate cancer: from prostate biopsy to radical prostatectomy. Mol Clin Oncol.

[3] Byun SS, Lee S, Lee SE, Lee E, Seo SI, Lee HM (2012). Recent changes in the clinicopathologic features of Korean men with prostate cancer: a comparison with Western populations. Yonsei Med J.

[4] Czyz J, Szpak K, Madeja Z (2012). The role of connexins in prostate cancer promotion and progression. Nat Rev Urol.

[5] Denmeade SR, Isaacs JT (2002). A history of prostate cancer treatment. Nat Rev Cancer.

[6] Siegel RL, Miller KD, Jemal A (2015). Cancer statistics. CA Cancer J Clin.

[7] Gan SS, Ye JQ, Wang L, Qu FJ, Chu CM, Tian YJ (2017). Inhibition of PCSK9 protects against radiation-induced damage of prostate cancer cells. Onco Targets Ther.

[8] Su XY, Zou X, Chen QZ, Zeng YH, Shao Y, He BC (2017). Follicle-stimulating hormone beta-subunit potentiates bone morphogenetic protein 9-induced osteogenic differentiation in mouse embryonic fibroblasts. J Cell Biochem.

[9] Guan YX, Zhang MZ, Chen XZ, Zhang Q, Liu SZ, Zhang YL, Lnc (2018). RNA SNHG20 participated in proliferation, invasion, and migration of breast cancer cells via miR-495. J Cell Biochem.

[10] Li Y, Shan Z, Yang B, Yang D, Men C, Cui Y (2018). LncRNA HULC promotes epithelial and smooth-muscle-like differentiation of adipose-derived stem cells by upregulation of BMP9. Pharmazie.

[11] Wang RN, Green J, Wang Z, Deng Y, Qiao M, Peabody M (2014). Bone morphogenetic protein (BMP) signaling in development and human diseases. Genes Dis.

[12] Wang Y, Feng Q, Ji C, Liu X, Li L, Luo J (2017). RUNX3 plays an important role in mediating the BMP9-induced osteogenic differentiation of mesenchymal stem cells. Int J Mol Med.

[13] Hao H, Chen H, Xie L, Liu H, Wang D (2021). LncRNA KCNQ1OT1 promotes proliferation, invasion and metastasis of prostate cancer by regulating miR-211-5p/CHI3L1 pathway. Onco Targets Ther.

[14] Gu P, Chen X, Xie R, Han J, Xie W, Wang B (2017). lncRNA HOXD-AS1 regulates proliferation and chemo-resistance of castration-resistant prostate cancer via recruiting WDR5. Mol Ther.

[15] Xu B, Mei J, Ji W, Bian Z, Jiao J, Sun J (2020). LncRNA SNHG3, a potential oncogene in human cancers. Cancer Cell Int.

[16] Wan Q, Tang M, Sun SL, Hu J, Sun ZJ, Fang YT (2021). SNHG3 promotes migration, invasion, and epithelial–mesenchymal transition of breast cancer cells through the miR-186-5p/ZEB1 axis. Am J Transl Res.

[17] Zhao S, Gao X, Zhong C, Li Y, Wang M, Zang S (2020). SNHG3 knockdown suppresses proliferation, migration and invasion, and promotes apoptosis in non-small cell lung cancer through regulating miR-216a/ZEB1 axis. Onco Targets Ther.

[18] Gueant JL, Oussalah A, Zgheib R, Siblini Y, Hsu SB, Namour F (2020). Genetic, epigenetic and genomic mechanisms of methionine dependency of cancer and tumor-initiating cells: what could we learn from folate and methionine cycles. Biochimie.

[19] Lien EC, Ghisolfi L, Geck RC, Asara JM, Toker A (2017). Oncogenic PI3K promotes methionine dependency in breast cancer cells through the cystine-glutamate antiporter xCT. Sci Signal.

[20] Poirson-Bichat F, Gonfalone G, Bras-Goncalves RA, Dutrillaux B, Poupon MF (1997). Growth of methionine-dependent human prostate cancer (PC-3) is inhibited by ethionine combined with methionine starvation. Br J Cancer.

[21] Sorin M, Watkins D, Gilfix BM, Rosenblatt DS (2021). Methionine dependence in tumor cells: the potential role of cobalamin and MMACHC. Mol Genet Metab.

[22] Chaturvedi S, Hoffman RM, Bertino JR (2018). Exploiting methionine restriction for cancer treatment. Biochem Pharmacol.

[23] Breillout F, Hadida F, Echinard-Garin P, Lascaux V, Poupon MF (1987). Decreased rat rhabdomyosarcoma pulmonary metastases in response to a low methionine diet. Anticancer Res.

[24] Guo H, Lishko VK, Herrera H, Groce A, Kubota T, Hoffman RM (1993). Therapeutic tumor-specific cell cycle block induced by methionine starvation in vivo. Cancer Res.

[25] Hoshiya Y, Guo H, Kubota T, Inada T, Asanuma F, Yamada Y (1995). Human tumors are methionine dependent in vivo. Anticancer Res.

[26] Komninou D, Leutzinger Y, Reddy BS, Richie JP (2006). Jr. Methionine restriction inhibits colon carcinogenesis. Nutr Cancer.

[27] Tan Y, Xu M, Guo H, Sun X, Kubota T, Hoffman RM (1996). Anticancer efficacy of methioninase in vivo. Anticancer Res.

[28] Tan Y, Xu M, Tan X, Tan X, Wang X, Saikawa Y (1997). Overexpression and large-scale production of recombinant l-methionine-alpha-deamino-gamma-mercaptomethane-lyase for novel anticancer therapy. Protein Expr Purif.

[29] Yang Z, Wang J, Yoshioka T, Li B, Lu Q, Li S (2004). Pharmacokinetics, methionine depletion, and antigenicity of recombinant methioninase in primates. Clin Cancer Res.

[30] Stern PH, Hoffman RM (1986). Enhanced in vitro selective toxicity of chemotherapeutic agents for human cancer cells based on a metabolic defect. J Natl Cancer Inst.

[31] Durando X, Thivat E, Farges MC, Cellarier E, D’Incan M, Demidem A (2008). Optimal methionine-free diet duration for nitrourea treatment: a phase I clinical trial. Nutr Cancer.

[32] Durando X, Farges MC, Buc E, Abrial C, Petorin-Lesens C, Gillet B (2010). Dietary methionine restriction with FOLFOX regimen as first line therapy of metastatic colorectal cancer: a feasibility study. Oncology.

[33] Sugiyama A, Ohta T, Obata M, Takahashi K, Seino M, Nagase S (2020). xCT inhibitor sulfasalazine depletes paclitaxel-resistant tumor cells through ferroptosis in uterine serous carcinoma. Oncol Lett.

[34] Wang Y, Sun L, Wang L, Liu Z, Li Q, Yao B (2018). Long non-coding RNA DSCR8 acts as a molecular sponge for miR-485-5p to activate Wnt/beta-catenin signal pathway in hepatocellular carcinoma. Cell Death Dis.

[35] Huang W, Tian Y, Dong S, Cha Y, Li J, Guo X (2017). The long non-coding RNA SNHG3 functions as a competing endogenous RNA to promote malignant development of colorectal cancer. Oncol Rep.

[36] Wang L, Su K, Wu H, Li J, Song D (2019). LncRNA SNHG3 regulates laryngeal carcinoma proliferation and migration by modulating the miR-384/WEE1 axis. Life Sci.

[37] Zheng S, Jiang F, Ge D, Tang J, Chen H, Yang J (2019). LncRNA SNHG3/miRNA-151a-3p/RAB22A axis regulates invasion and migration of osteosarcoma. Biomed Pharmacother.

[38] Zhang H, Wei N, Zhang W, Shen L, Ding R, Li Q (2020). lncRNA SNHG3 promotes breast cancer progression by acting as a miR326 sponge. Oncol Rep.

[39] Chan JJ, Tay Y (2018). Noncoding RNA:RNA regulatory networks in cancer. Int J Mol Sci.

[40] Li T, Xing Y, Yang F, Sun Y, Zhang S, Wang Q (2020). LncRNA SNHG3 sponges miR-577 to up-regulate SMURF1 expression in prostate cancer. Cancer Med.

[41] Sun LB, Zhao SF, Zhu JJ, Han Y, Shan TD (2020). Long noncoding RNA UCID sponges miR1523p to promote colorectal cancer cell migration and invasion via the Wnt/betacatenin signaling pathway. Oncol Rep.

[42] Ma P, Li L, Liu F, Zhao Q (2020). HNF1A-induced lncRNA HCG18 facilitates gastric cancer progression by upregulating DNAJB12 via miR-152-3p. Onco Targets Ther.

[43] Shi J, Zhang Y, Qin B, Wang Y, Zhu X (2019). Long non-coding RNA LINC00174 promotes glycolysis and tumor progression by regulating miR-152-3p/SLC2A1 axis in glioma. J Exp Clin Cancer Res.

[44] Feng F, Liu H, Chen A, Xia Q, Zhao Y, Jin X (2019). miR-148-3p and miR-152-3p synergistically regulate prostate cancer progression via repressing KLF4. J Cell Biochem.

[45] Tao LJ, Pan XY, Wang JW, Zhang L, Tao LS, Liang CZ (2021). Circular RNA circANKS1B acts as a sponge for miR-152-3p and promotes prostate cancer progression by upregulating TGF-alpha expression. Prostate.

[46] Ramalho-Carvalho J, Goncalves CS, Graca I, Bidarra D, Pereira-Silva E, Salta S (2018). A multiplatform approach identifies miR-152-3p as a common epigenetically regulated onco-suppressor in prostate cancer targeting TMEM97. Clin Epigenet.

[47] Ji X, Qian J, Rahman SMJ, Siska PJ, Zou Y, Harris BK (2018). xCT (SLC7A11)-mediated metabolic reprogramming promotes non-small cell lung cancer progression. Oncogene.

[48] Wu P, Li C, Ye DM, Yu K, Li Y, Tang H (2021). Circular RNA circEPSTI1 accelerates cervical cancer progression via miR-375/409-3P/515-5p-SLC7A11 axis. Aging.

[49] Ma J, Kan Z (2021). Circular RNA circ_0008274 enhances the malignant progression of papillary thyroid carcinoma via modulating solute carrier family 7 member 11 by sponging miR-154-3p. Endocr J.

[50] Fu D, Wang C, Yu L, Yu R (2021). Induction of ferroptosis by ATF3 elevation alleviates cisplatin resistance in gastric cancer by restraining Nrf2/Keap1/xCT signaling. Cell Mol Biol Lett.

[51] Cavuoto P, Fenech MF (2012). A review of methionine dependency and the role of methionine restriction in cancer growth control and life-span extension. Cancer Treat Rev.

[52] Wanders D, Hobson K, Ji X (2020). Methionine restriction and cancer biology. Nutrients.

[53] Mavrakis KJ, McDonald ER, Schlabach MR, Billy E, Hoffman GR, deWeck A (2016). Disordered methionine metabolism in MTAP/CDKN2A-deleted cancers leads to dependence on PRMT5. Science.

[54] Chen K, Liu H, Liu Z, Luo S, Patz EF, Moorman PG (2019). Genetic variants in RUNX3, AMD1 and MSRA in the methionine metabolic pathway and survival in nonsmall cell lung cancer patients. Int J Cancer.

[55] Lu S, Epner DE (2000). Molecular mechanisms of cell cycle block by methionine restriction in human prostate cancer cells. Nutr Cancer.

[56] Cellarier E, Durando X, Vasson MP, Farges MC, Demiden A, Maurizis JC (2003). Methionine dependency and cancer treatment. Cancer Treat Rev.

[57] Pena-Ramos J, Hinchliffe TA, Costa M, Sanchez-Redondo S (2021). Suspected sulfasalazine-induced acute hepatic necrosis in a dog after one year of continuous treatment. Vet Re Case Rep.

