# Supplementary material for: SNHG3 could promote prostate cancer progression through reducing methionine dependence of PCa cells
Source: Cell Mol Biol Lett. 2022 Feb 5;27:13. doi: 10.1186/s11658-022-00313-z (PMC8903624; doi:10.1186/s11658-022-00313-z)
Supplement: Supplementary file 1 — Additional file 1: Table S1. Correlations between SNHG3 expression and clinicopathological characteristics in prostate cancer. Table S2. Correlations between miR-152-3p expression and clinicopathological characteristics in prostate cancer. Table S3. Correlations between SLC7A11 expression and clinicopathological characteristics in prostate cancer. [file 11658_2022_313_MOESM1_ESM.docx]

**Table S1. Correlations between SNHG3 expression and clinicopathological characteristics in prostate cancer**

| Characteristics | n | Low expression (%) | High expression (%) | P-value |
| --- | --- | --- | --- | --- |
| Age (years) |  |  |  |  |
| ≤65 | 32 | 16(50.00%) | 16(50.00%) | 1.000 |
| >65> | 48 | 24(50.00%) | 24(50.00%) |  |
| Clinical T stage |  |  |  |  |
| T1–T2 | 36 | 23(63.88%) | 13(36.11%) | 0.025 |
| T3–T4 | 44 | 17(38.63%) | 27(61.36%) |  |
| Lymph node involvement |  |  |  |  |
| No | 63 | 36(57.14%) | 27(42.85%) | 0.014 |
| Yes | 17 | 4(23.52%) | 13(76.47%) |  |
| Distant metastasis |  |  |  |  |
| No | 65 | 37(56.92%) | 28(43.07%) | 0.010 |
| Yes | 15 | 3(20.00%) | 12(80.00%) |  |
| Gleason score |  |  |  |  |
| ≤8 | 35 | 23(65.71%) | 12(34.28%) | 0.013 |
| >8 | 45 | 17(37.77%) | 28(62.22%) |  |
| Serum PSA (ng/mL） |  |  |  |  |
| ≤20 | 31 | 16(51.61%) | 15(48.38%) | 0.818 |
| >20 | 49 | 24(48.97%) | 25(51.02%) |  |

**Table S2 Correlations between miR-152-3p expression and clinicopathological characteristics in prostate cancer**

| Characteristics | n | Low expression (%) | High expression (%) | P-value |
| --- | --- | --- | --- | --- |
| Age (years) |  |  |  |  |
| ≤65 | 32 | 14(43.75%) | 18(56.25%) | 0.361 |
|  | 48 | 26(54.16%) | 22(45.83%) |  |
| Clinical T stage |  |  |  |  |
| T1–T2 | 36 | 14(38.88%) | 22(61.11%) | 0.072 |
| T3–T4 | 44 | 26(59.09%) | 18(40.90%) |  |
| Lymph node involvement |  |  |  |  |
| No | 63 | 26(41.26%) | 37(58.73%) | 0.003 |
| Yes | 17 | 14(82.35%) | 3(17.64%) |  |
| Distant metastasis |  |  |  |  |
| No | 65 | 27(41.53%) | 38(58.46%) | 0.002 |
| Yes | 15 | 13(86.66%) | 2(13.33%) |  |
| Gleason score |  |  |  |  |
| ≤8 | 35 | 11(31.42%) | 24(68.57%) | 0.003 |
|  | 45 | 29(64.44%) | 16(35.55%) |  |
| Serum PSA (ng/mL） |  |  |  |  |
| ≤20 | 31 | 17(54.83%) | 14(45.16%) | 0.491 |
|  | 49 | 23(46.93%) | 26(53.06%) |  |

**Table S3. Correlations between SLC7A11 expression and clinicopathological characteristics in prostate cancer**

| Characteristics | n | Low expression (%) | High expression (%) | P-value |
| --- | --- | --- | --- | --- |
| Age (years) |  |  |  |  |
| ≤65 | 32 | 17(53.12%) | 15(46.87%) | 0.648 |
|  | 48 | 23(47.91%) | 25(52.08%) |  |
| Clinical T stage |  |  |  |  |
| T1–T2 | 36 | 21(58.33%) | 15(41.66%) | 0.178 |
| T3–T4 | 44 | 19(43.18%) | 25(56.81%) |  |
| Lymph node involvement |  |  |  |  |
| No | 63 | 36(57.14%) | 27(42.85%) | 0.014 |
| Yes | 17 | 4(23.52%) | 13(76.47%) |  |
| Distant metastasis |  |  |  |  |
| No | 65 | 35(53.84%) | 30(46.15%) | 0.152 |
| Yes | 15 | 5(33.33%) | 10(66.66%) |  |
| Gleason score |  |  |  |  |
| ≤8 | 35 | 24(68.57%) | 11(31.42%) | 0.003 |
|  | 45 | 16(35.55%) | 29(64.44%) |  |
| Serum PSA (ng/mL） |  |  |  |  |
| ≤20 | 31 | 15(48.38%) | 16(51.61%) | 0.818 |
|  | 49 | 25(51.02%) | 24(48.97%) |  |
